# Gene Deletions and Prognostic Values in B-Linage Acute Lymphoblastic Leukemia

**DOI:** 10.3389/fonc.2021.677034

**Published:** 2021-06-02

**Authors:** Qiuyun Fang, Yang Song, Xiaoyuan Gong, Jun Wang, Qinghua Li, Kaiqi Liu, Yahui Feng, Qishan Hao, Yan Li, Hui Wei, Guangji Zhang, Yuntao Liu, Benfa Gong, Ying Wang, Chunlin Zhou, Dong Lin, Bingcheng Liu, Shuning Wei, Runxia Gu, Yingchang Mi, Jianxiang Wang

**Affiliations:** State Key Laboratory of Experimental Hematology, National Clinical Research Center for Blood Disease, Institute of Hematology and Blood Diseases Hospital, Chinese Academy of Medical Sciences, Tianjin, China

**Keywords:** adult, acute lymphoblastic leukemia, molecular abnormalities, gene deletions, prognostic analysis

## Abstract

Although pediatric-like treatment regimen has remarkably improved the survival rates of adults with acute lymphoblastic leukemia (ALL), the outcome of some adult patients is still poor owing to adverse genetic features. These molecular abnormalities, especially gene deletions, may be considered for the prognosis assessment for adult patients with ALL. In this study, using multiplex ligation-dependent probe amplification (MLPA) method, gene deletions were analyzed in from 211 adult B-ALL patients treated in our center. The data showed that 68.2% (144/211) adult B-ALL patients carried gene deletions, and the frequency is much higher in Ph^+^B-ALL patients. *IKZF1* gene deletion is the most common gene deletion in adult B-ALL, followed by *CDKN2A/B* deletion. In Ph^-^B-ALL patients, the overall survival of patients with gene deletions is inferior to that of patients without any gene deletions. More obviously, patients with *IKZF1* or *CDKN2A/B* deletion had a worse prognosis, whereas, allogeneic hematopoietic stem cell transplantation could improve OS in patients with *IKZF1* deletion, but not in patients with CDKN2A/B deletion. Moreover, the outcome of Ph^-^B-ALL patients with double deletion of *IKZF1*and *CDKN2A/B* may be much worse than that of patients with *IKZF1* or *CDKN2A/B* alone. Minimal residual disease (MRD) was also analyzed together with gene deletions and demonstrated that gene deletions have a negative impact on survival only in MRD positive Ph^-^B-ALL patients. In conclusion, gene deletions are closely related with the prognosis of adult Ph^-^B-ALL patients.

## Introduction

Acute lymphoblastic leukemia (ALL) is a malignant transformation and proliferation of lymphoid progenitor cells in the bone marrow, blood and extramedullary sites, with more than 75% originating from B-linage (B-ALL) (1). Recent years have witnessed a significant increase of >90% in survival rates in pediatric ALL (2); however, the long-term outcome of adult ALL is much inferior to that of pediatric B-ALL, including many low-risk patients with genetic heterogeneities and varied intensity of the treatments (3). Moreover, unlike adult acute myeloid leukemia, there is a lack of studies on adult ALL’s molecular prognostic factors and a lack of consensus on molecular abnormalities in adult ALL risk stratification. Hence, the discovery of robust and assertive prognostic indicators is an urgent requirement for personalized clinical decisions.

Thus, the role of gene deletions in hematological malignancies and their prognostic values have garnered increasing research interest. A large number of studies have focused on extrapolating well-established molecular signatures in the prognostic assessment of pediatric ALL to adult ALL. For example, *IKZF1* deletion, a hallmark of high-risk pediatric ALL, is related to poor prognosis in adult ALL (4–10). This phenomenon was consistent with our previous study, wherein *IKZF1* deletion was associated with poor survival in adult B-ALL patients (11, 12).

Stanulla et al. (13) found that *IKZF1* deletion in patients those without *ERG* deletion was often accompanied by other gene deletions (*CDKN2A/2B, PAX5*, and *PAR1*), which was defined as *IKZF1^plus^* gene abnormality. The study also demonstrated that *IKZF1^plus^* presents a poor prognostic profile in pediatric B-ALL. However, large discrepancies are noted with respect to concomitant abnormalities of *IKZF1* deletion and the prognostic value in different reports (14–19), and whether *IKZF1^plus^* achieves an equally superior prognostic performance in all B-ALL patients remains unknown.

In this study, multiplex ligation-dependent probe amplification (MLPA) method was used to detect 13 gene deletions in 211 adult B-ALL patients who were diagnosed and treated in our center from October 2009 to December 2018. The characteristics of patients with different gene deletions and gene deletions’ prognostic impact stratified by disease subgroups were analyzed.

In the current study, we revealed the gene deletion frequency of *IKZF1*, *CDKN2A/B*, *I&C* and other genes in Chinese adult B-ALL patients using MLPA, and established gene deletions as prognostic indicators for these patients across various sub-groups. These findings shed light on the roles of gene deletions in the initiation and progression of B-ALL, highlight the importance of shifting from individual risk factors to integrated prognostic profiles, and open an avenue towards the development of personalized treatment strategies for ALL.

## Materials and Methods

### Patients and Samples

The present study included 211 patients with newly diagnosed B-ALL in a prospective single arm clinical trial (ChiCTR-TNC-09000397) (20) at the Leukemia Center in the Institute of Hematology and Blood Diseases Hospital (Tianjin, China) between October 2009 and December 2018. All the experiments were performed in accordance with the Helsinki Declaration, and the study was approved by the institutional ethics committee. Informed consent was obtained from all patients.

All 211 patients were diagnosed by MICM mode, which included morphology, immunology, and molecular and genetic analysis. The median percentage of bone marrow leukemia blasts was 88.5% (range: 22.5-99.5%; Ph^-^B-ALL: 89%, 22.5-95%, Ph^+^B-ALL: 86%, 23.5-99.5%, 2 patients with blasts percentage: 20-30%). Patients with Ph^-^B-ALL were treated with the therapy schedule, according to the trial protocol. The induction regimen is VDCLP, and after reaching complete remission (CR), all patients received early intensive, late consolidation, and maintenance treatment. Ph^+^B-ALL patients were treated in the same manner with L-asparaginase omitted and a TKI (Imatinib or Dasatinib) added from the 8th day of induction chemotherapy. Among 211 patients, the CR rate was 95.7% (202/211). Nine (4.3%, Ph^-^B-ALL:7, Ph^+^B-ALL:2) patients did not achieve CR: 4 (1.9%) died of complications in induction therapy, 3 died of disease without remission, and data could not be evaluated in 2 patients; 8 patients harbored gene deletions, and 1 patient did not carry gene deletions (**Supplementary Data 1**). The data of minimal residual disease (MRD, detected using flow cytometry) after induction treatment and at 3 months post-initial treatment were obtained in 172 patients. The cutoff of flow cytometry result was 10^-4^. Patients with flow cytometry result <10^-4^ at both test points were considered as MRD-negative. Patients with one or both results ≥10^-4^ were considered as MRD-positive. High risk patients (according to NCCN risk stratification criteria), and patients with MRD positive at any test points were recommended to receive allo-HSCT. The time-point of HSCT was after 2 cycles of consolidation therapy.

The median follow-up time was 23.59 (range, 0.53–123.96) months, and the rate of loss to follow-up was 3.8% (8/211).

### MLPA

Bone marrow mononuclear cells of the patients were collected at the time of diagnosis, and DNA was extracted using the QIAamp DNA Blood Mini kit (catalog #51104; Qiagen GmbH, Hilden, Germany), according to the manufacturer’s protocols. The MLPA assay was conducted using the SALSA MLPA P335 ALL-IKZF1 Probe Mix Kit (MRC Holland, Amsterdam, The Netherlands) to detect the deletion of genes related to ALL pathogenesis, according to the manufacturer’s protocol (11). This assay contains 13 genes, including part or whole exons of *IKZF1*, *EBF1*, *CDKN2A/B, PAX5, ETV6, BTG1, RB1, SHOX-AREA, CRLF2, CSF2RA, IL3RA and P2RY8* genes. The products were subjected to fragment analysis on an ABI-3730 genetic analyzer (Applied Biosystems, Carlsbad, CA, USA), and the MLPA data were analyzed using Coffalyser^®^ software.

### Statistical Analysis

All statistical analyses were performed using SPSS 21.0 software (IBM Co., Armonk, NY, USA). The overall survival (OS) was defined as the time from diagnosis to the time of death (from any cause) or last follow-up, and the relapse-free survival (RFS) was defined as the time from achieving CR to relapse or death or the date of the last follow-up in CR. OS and RFS were evaluated by Kaplan-Meier analysis and compared by the log-rank test. Cox proportional hazard regression models were used to identify the prognostic factors. The values of the specific deleted gene were adjusted by age, white blood cell (WBC) count, MRD, and the allogeneic hematopoietic stem cell transplantation (allo-HSCT) and included in the model as a time-varying covariate. The other comparisons were performed using the chi-square or Fisher’s exact test. P<0.05 indicated statistical significance.

## Results

### Clinical Characteristics of Patients

The cohort consisted of 211patients, including 118 males, with a median age of 32 (14–69) years. The median WBC count was 22.1 (0.57–81.2) ×10^9^/L. Among all patients, 85 cases were Ph^+^ALL, and 126 cases were Ph^-^ALL (5/120 patients harbored MLL rearrangement). Based on the guidelines by Hoelzer et al. (11), 45 cases were stratified in the standard-risk (SR) group, and 166 cases were in the high-risk (HR) group. A total of 100 patients received allo-HSCT (Ph^+^B-ALL: 50, Ph^-^B-ALL: 50), 6 Ph^-^B-ALL patients received HSCT at the second complete remission (**Supplementary Data 1**), while 111 patients did not receive HSCT (Ph^+^B-ALL: 35, Ph^-^B-ALL: 76).

According to the MRD data, 172 patients were classified into two groups: 72 patients were Ph^+^B-ALL patients (MRD-negative: 48, MRD-positive: 24); 100 were Ph^-^B-ALL patients (MRD-negative: 62, MRD-positive: 38).

### Gene Deletions

Among the 211 patients, 144 (68.2%) were found to have gene deletions. Among those with gene deletions, 70 (70/85, 82.4%) were Ph^+^B-ALL patients, 74 (74/126, 58.7%) were Ph^-^B-ALL patients (P<0.01). Subsequently, 75/144 patients underwent allo-HSCT (Ph^+^B-ALL: 42, Ph^-^B-ALL: 33). In the group of patients without any gene deletions, 15 (15/85, 17.6%) were Ph^+^B-ALL, 52 (52/126, 41.3%) were Ph^-^B-ALL, and 25 underwent HSCT (Ph ^+^ group: 8, Ph^-^ group: 17). Among the patients with MRD, 116 constituted the gene deletion group (MRD-negative: 59.5%, 69/116; MRD-positive: 40.5%, 47/116) and 56 cases were no-gene deletion group (MRD-negative: 73.2%, 41/56; MRD-positive: 26.8%, 15/56) (**Table 1**).

**Table 1:** Patient characteristics grouped by gene deletion status.

	Gene deletion (N=144)	No Gene deletion (N=67)	P value
Gender			
Male	82(56.9%)	36(53.7%)	0.662
Female	62(43.1%)	31(46.3%)	
Age	14-69	14-64	
Median	32	30	0.37
WBC	0.57-457×10^9^/L	0.95-481×10^9^/L	
Median	28.54×10^9^/L	13.1×10^9^/L	0.58
Risk stratification			
SR (N=45)	24 (53.3%)	21 (46.7%)	<0.01
HR (N=166)	120 (72.3%)	46 (27.7%)	
Ph			
Ph+ (N=85)	70 (82.4%)	15 (17.6%)	<0.01
Ph- (N=126)	74 (58.7%)	52 (41.3%)	
MRD			
(-)	69/116(59.5%)	41/56(73.2%)	<0.01
(+)	47/116(40.5%)	15/56(26.8%)	
CR Rate (N=202)	136/144(94.4%)	66/67(98.5%)	0.174
HSCT			
Yes	75 (52.1%)	25 (37.3%)	0.045
No	69 (47.9%)	42 (62.7%)	

HSCT, hematologic stem cell transplantation; MRD, minimal residual disease; Ph^-^, Ph chromosome negative; Ph^+^, Ph chromosome positive; SR, standard risk; WBC, White blood cell.

#### IKZF1 Gene Deletion

**Figure 1:**
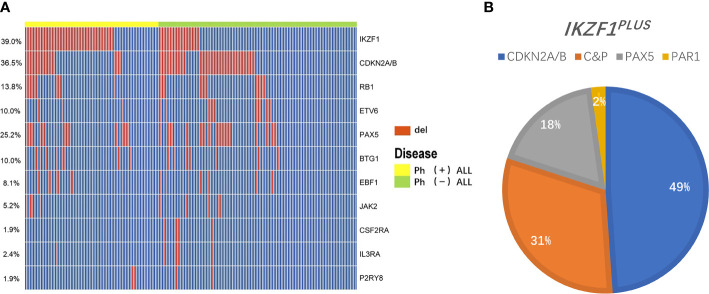
Overview of gene deletions detected by MLPA. **(A)** A heatmap showing the prevalence of main gene deletions in 211 adult B-ALL. The red bar represents the patients carried the gene deletion, the blue bar represents the patients didn’t carry the gene deletion. **(B)** A pie chart depicting the proportion of specific types of *IKZF1^PLUS^* deletion. Different color represents different gene deletions in concurrence with *IKZF1* deletion: Blue, CDKN2A/B (49%); Orange, C&P (CDKN2A/B and PAX5, 31%); Gray, PAX5 (18%); and Yellow, PAR1 (2%).

*IKZF1* gene deletion was detected in 82/211 (38.9%) patients (**Figure 1A**). The deletion rate of *IKZF1* in the Ph^+^B-ALL group (N=56) was significantly higher than that of the Ph^-^B-ALL group (N=26) (65.9% *vs*. 20.6%, P<0.01). Different types of intragenic deletions of *IKZF1* had been observed, resulting in a “loss-of-function” or “dominant-negative” isoforms according to Kobitzsch’s research (9). We analyzed the deletion types of *IKZF1*, 14 cases (17.1%) were whole gene deletion type, 33 cases (40.2%) were dominant negative type, and 29 (35.4%) cases were loss of function type.

48/82 patients with *IKZF1* deletion underwent HSCT, while the remaining 34 patients did not receive HSCT. MRD results were obtained in 66 patients with *IKZF1* deletion, 36 patients were MRD-negative, and 30 were MRD-positive. Patients with *IKZF1* deletion showed higher MRD positive rate than *IKZF1* wild-type patients (P=0.043) (**Table 2** and **Supplementary Table 1**).

**Table 2:** Prevalence of various gene deletions in different patient groups.

	B-ALL (211)	Ph^+^ (85)	Ph^-^ (126)	P value	Ph^+^		Ph^-^				P value
					HSCT(50)	NHSCT(35)	HSCT(50)	NHSCT(76)	SR (44)	HR(82)	
IKZF1	82	56	26	0.000	36	20	12	14	8	18	0.618
	38.9%	65.9%	20.3%		72%	57.1%	24%	18.4%	18.2%	22%	
I&C	36	19	17	0.093	11	8	6	11	6	11	0.972
	17.1%	22.4%	13.5%		22%	22.9%	12%	14.5%	13.6%	13.4%	
CDKN2A	71	21	50	0.024	11	10	25	25	17	33	0.86
	33.6%	24.7%	39.7%		22%	28.6%	50%	32.9%	38.6%	40.2%	
CDKN2B	60	19	41	0.131	11	8	21	20	14	27	0.899
	28.4%	22.4%	32%		22%	22.9%	42%	26.3%	31.8%	32.9%	
CDKN2A/B	77	24	53	0.041	13	11	26	27	17	36	0.568
	36.5%	28.2%	42.1%		26%	31.4%	52%	35.5	38.6%	43.9%	
PAX5	53	23	30	0.549	10	13	11	19	9	21	0.517
	25.1%	27.1%	23.8%		20%	37.1%	22%	0.25%	20.5%	25.6%	
ETV6	21	5	16	0.113	1	4	3	13	4	12	0.373
	9.9%	5.9%	12.7%		2%	11.4%	6%	17.1%	9.1%	14.6%	
RB1	29	14	15	0.322	10	4	5	10	6	9	0.867
	13.7%	16.5%	11.9%		20%	11.4%	10%	13.2%	13.6%	11%	
EBF1	17	10	7	0.097	5	5	4	3	2	5	0.717
	8.1%	11.8%	5.6%		10%	14.3%	8%	3.9%	4.5%	6.1%	
BTG1	21	10	11	0.447	5	5	4	7	1	10	0.06
	9.9%	11.8%	8.7%		10%	14.3%	8%	9.2%	2.3%	12.2%	

B-ALL, B-linage acute lymphoblastic leukemia; HSCT, hematologic stem cell transplantation; HR, high risk; NHSCT, non hematologic stem cell transplantation; Ph^-^, Ph chromosome negative; Ph^+^, Ph chromosome positive; I&C, IKZF1 and CDKN2A/B; SR, standard risk.

#### IKZF1 Deletion Companied With Other Gene Deletions

*IKZF1^PLUS^* was defined by Stanulla et al. (13) as *IKZF1* deletion accompanied by at least one gene deletion in *CDKN2A/B*, *PAX5*, *PAR1* in the absence of *ERG* deletion in pediatric B-ALL. In the current study, we did not assess *ERG* deletion and *P2RY8-CRLF2* fusion in the whole cohort, thus we analyzed patients who carried *IKZF1* deletion in combination with any of the other recurrent deletions (*IKZF1*+other deletion) instead of *IKZF1^PLUS^.* 45/211 (21.3%, Ph^+^B-ALL: 26; Ph^-^B-ALL: 19) patients had this kind deletion. The specific types of *IKZF1*+other deletion were as follows: 36 cases of *IKZF1* deletion combined with *CDKN2A/B* deletion (Ph^+^B-ALL: 19; Ph^-^B-ALL: 17), including 22 cases of single *CDKN2A/B* deletion. Among the 45 cases, 22 patients presented *IKZF1* and *PAX5* deletion simultaneously, 8 patients had single *PAX5* deletion; 4 cases had *IKZF1* and *PAR1* deletion, and 1 case had *PAR1* alone. Furthermore, 14 *IKZF1* deletion cases also showed *CDKN2A/B* and *PAX5* deletion together (**Figure 1B**). *IKZF1*+other deletions rate in Ph^+^B-ALL was higher than that in Ph^-^B-ALL (30.6% *vs*. 14.8%, P<0.01). Of the 45 patients with *IKZF1*+other deletions, 23 cases underwent HSCT, and 22 cases did not receive HSCT (**Table 2**). A total of 36 patients with *IKZF1*+other deletions obtained MRD results (**Supplementary Table 2**).

#### CDKN2A/B Gene Deletion

Among the 211 B-ALL patients, 77 (36.5%) had *CDKN2A/B* deletion. Among them, 71 (33.6%) had *CDKN2A* deletion, 60 (28.4%) had *CDKN2B* deletion, and 54 (25.6%) had both gene deletions (**Figure 1A**). The incidence of *CDKN2A/B* deletion in Ph^-^B-ALL patients (53/126, 42.1%) was significantly higher than that in Ph^+^B-ALL patients (24/85, 28.2%, P=0.041). Then, 39 patients with *CDKN2A/B* deletion underwent HSCT, the other 38 patients did not (**Table 2**). MRD results were obtained in 62 patients with *CDKN2A/B* deletion: 36 were MRD-negative, and 26 were MRD-positive (**Supplementary Table 2**).

Among 211 patients, 36 patients harbored *IKZF1 and CDKN2A/B* deletions simultaneously. Among them, 17 patients underwent HSCT.

#### PAX5 Gene Deletion

The deletion of *PAX5* gene was detected in 53 (25.1%) cases of 211 B-ALL patients, (Ph^+^B-ALL: 23, 27.1%, Ph^-^B-ALL: 30, 23.8%; P=0.549). No significant difference was observed in the incidence of *PAX5* gene deletion in different Ph chromosome and risk groups. Among them, 21 patients with *PAX5* deletion underwent HSCT (**Table 2** and **Supplementary Table 1**).

### Prognosis of Gene Deletion in Ph^-^B-ALL

#### The 2-Year OS and RFS in the Gene Deletion Group Were Significantly Worse Than Those With No Gene Deletion in Ph^-^B-ALL

**Figure 2:**
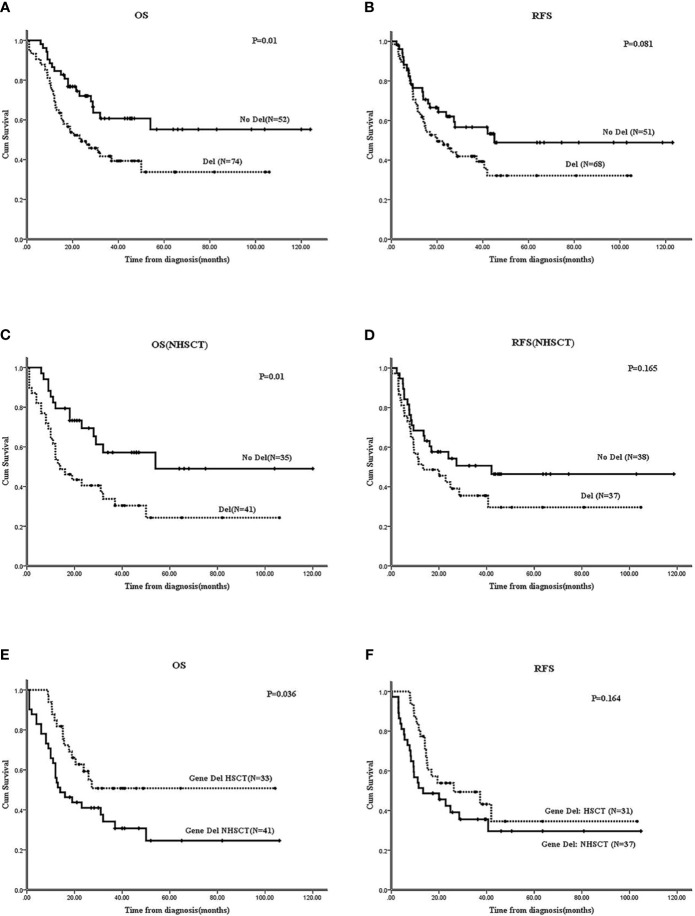
Effect of gene deletions on survivals of Ph^-^B-ALL patients by Kaplan-Meier plots. **(A, B)** The 2-year OS and RFS of Ph^-^B-ALL patients who carried gene deletion vs. none gene deletion. **(C, D)** The 2-year OS and RFS of Ph^-^B-ALL patients who carried gene deletion vs. none gene deletion in no HSCT group (NHSCT: No HSCT). **(E, F)** The 2-year OS and RFS of Ph^-^B-ALL patients with gene deletions who received HSCT vs. who didn’t receive HSCT. Note: In all the RFS analysis of this study, 6 Ph-B-ALL patients received HSCT at CR2, the 6 patients were analyzed in non-HSCT group, and 9 patients who didn’t receive CR were also excluded from RFS analysis.

The 3-year OS of 211 B-ALL patients was 43.2 ± 3.7%, and the 2-year RFS was 51.8 ± 3.6%. In 126 Ph^-^B-ALL patients, the 2-year OS was58.5 ± 3.5%, and the 3-year RFS was 50.1 ± 3.5% (**Supplementary Figure 1**). Among the 126 Ph^-^B-ALL patients, 52 cases had no gene deletion (17 cases received HSCT, 32.7%), and 74 cases presented gene deletion (33 cases underwent HSCT, 44.6%). The survival of patients without any gene deletions was significantly better than that of patients with gene deletions (2-year OS: 72.1 ± 6.4% *vs*. 49.2 ± 5.9%, P=0.01; 2-year RFS:62 ± 6.9% *vs*. 47.8 ± 6.1%, P=0.081; **Figures 2A, B**). In patients who did not receive HSCT, the 2-year OS and RFS in no-gene deletion group (N=35) were better than those in the gene deletion group (N=41) (OS: 69.4 ± 8.1% *vs*. 40.5 ± 7.9%, P=0.01; RFS: 62.4 ± 8.3% *vs*. 54.3 ± 8.3%, P=0.165) (**Figures 2C, D**). Allo-HSCT improves the OS of patients with gene deletions; the 2-year OS of patients who received HSCT (N=33) *vs*. patients who did not receive HSCT (N=41) was 59.3 ± 8.8% *vs*. 43.8 ± 7.8% (P=0.036), and the 2-year RFS: 53.9 ± 9.1% *vs*. 39.1 ± 8.3%, P=0.164 (**Figures 2E, F**).

#### In Ph-B-ALL, IKZF1 Deletion Was an Inferior Factor in Those Who Didn’t Receive HSCT

**Figure 3:**
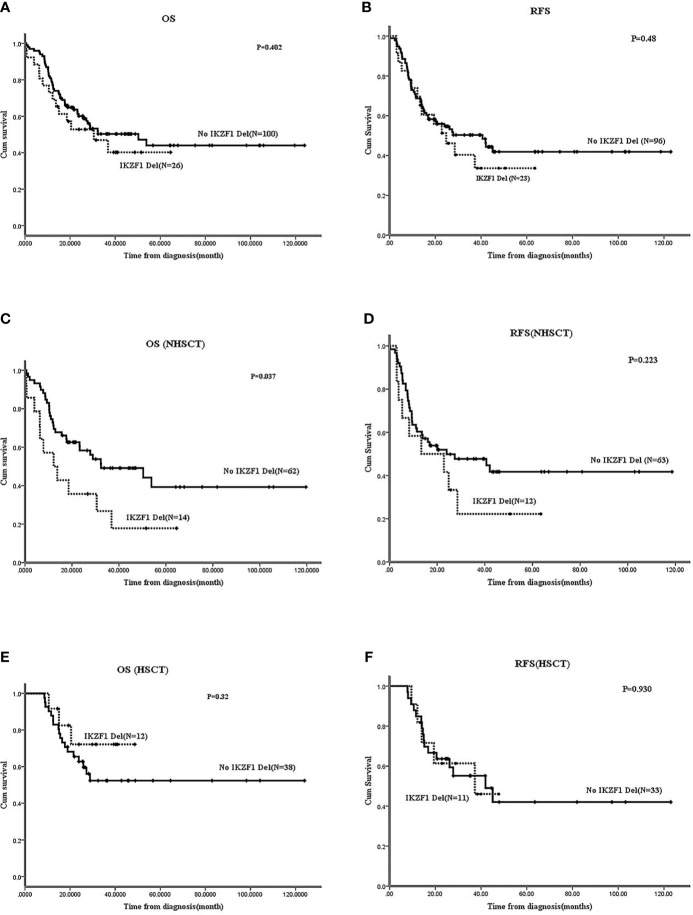
Effect of *IKZF1* deletion on survivals of Ph-B-ALL patients by Kaplan-Meier plots. **(A, B)** The 2-year OS and RFS of Ph^-^B-ALL patients who carried *IKZF1* deletion vs. no *IKZF1* deletion. **(C, D)** The 2-year OS and RFS of Ph^-^B-ALL patients who carried *IKZF1* deletion vs. no *IKZF1* deletion in no HSCT group. **(E, F)** The 2-year OS and RFS of Ph^-^B-ALL patients who carried *IKZF1* deletion vs. no *IKZF1* deletion in HSCT group.

In Ph^-^B-ALL, no significant differences were observed in OS and RFS between patients with *IKZF1* deletion and those without *IKZF1* deletion (2-year OS: 52.8 ± 10% *vs*. 60.1 ± 5%, P=0.402; 2-year RFS: 51.2 ± 10.6% *vs*. 54.6 ± 5.1%, P=0.48) (**Figures 3A, B**). No survival differences were found between different *IKZF1* deletion types and those patients without the deletion (data not shown).

Further analysis was carried out according to whether the patients received allo-HSCT. In patients without HSCT, the 2-year OS and 2-year RFS of patients with *IKZF1* deletion (N=14) were worse than those without *IKZF1* deletion (N=62) (OS: 17.9 ± 11% *vs*. 62.6 ± 6.3%, P=0.037; RFS: 41.7 ± 14.2% *vs*. 49.9 ± 6.4%, P=0.223) (**Figures 3C, D**). In patients with HSCT, no difference was found in the 2-year OS or RFS between patients with *IKZF1* deletion (N=12) and those without *IKZF1* deletion (N=38) (2-year OS: 72.2 ± 13.8% *vs*. 62.8 ± 7.7%, P=0.32; 2-year RFS: 61.4 ± 15.3% *vs*. 63.6 ± 8.4%, P=0.93) (**Figures 3E, F**). Because of the small sample size, the survival analysis of *IKZF1+*other deletions was not performed in Ph^-^B-ALL cohort.

#### CDKN2A/B Deletion Had Crucial Prognostic Significance in Ph^-^B-ALL

**Figure 4:**
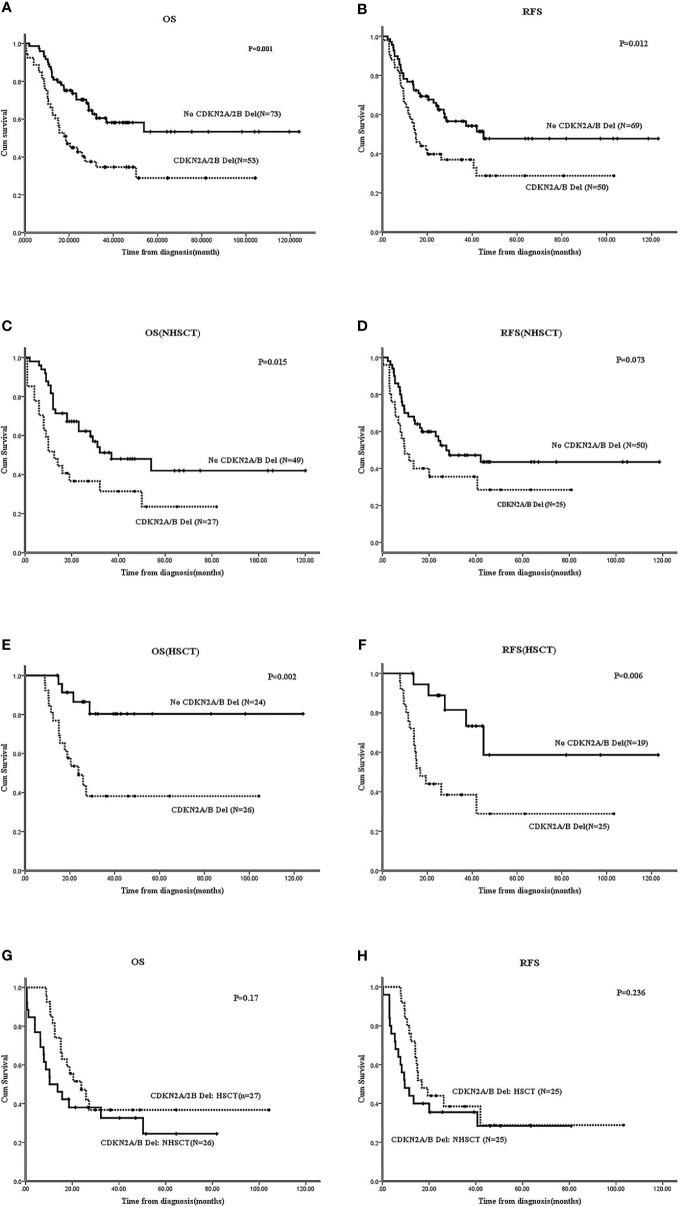
Effect of *CDKN2A/B* deletion on survivals of Ph-B-ALL patients by Kaplan-Meier plots. **(A, B)** The 2-year OS and RFS of Ph-B-ALL patients who carried *CDKN2A/B* deletion vs. no *CDKN2A/B* deletion. **(C, D)** The 2-year OS and RFS of Ph-B-ALL patients who carried *CDKN2A/B* deletion vs. no *CDKN2A/B* deletion in no HSCT group. **(E, F)** The 2-year OS and RFS of Ph-B-ALL patients who carried *CDKN2A/B* deletion vs. no *CDKN2A/B* deletion in HSCT group. **(G, H)** The 2-year OS and RFS of Ph-B-ALL patients with *CDKN2A/B* deletion who received HSCT vs. no HSCT.

In Ph^-^B-ALL patients, 53/126 (42.1%) cases carried *CDKN2A/B* deletion; the survival of these patients was inferior to those without *CDKN2A/B* deletion (N=73). The 2-year OS: 54.7 ± 6.8% *vs*. 75.2 ± 5.1%, P=0.001; 2-year RFS: 39.8 ± 6.9% *vs*. 59.9 ± 6.9%, P=0.012 (**Figures 4A, B**). In the non-HSCT group, *CDKN2A/B* deletion patients (N=27) *vs*. no *CDKN2A/B* deletion patients (N=49) showed that the 2-year OS was 32.6 ± 9.8% *vs*. 62.2 ± 7.1% (P=0.015) and the 2-year RFS was 35.6 ± 9.7% *vs*. 59.9 ± 6.9% (P=0.073) (**Figures 4C, D**). In the HSCT group, *CDKN2A/B* deletion patients (N=24) *vs*. no *CDKN2A/B* deletion patients (N=26) showed that the 2-year OS was 38.2 ± 10.3% *vs*. 80.3 ± 9% (P=0.002) and the 2-year RFS was 44 ± 9.9% *vs*. 88.9 ± 7.4% (P=0.006) (**Figures 4E, F**). HSCT failed to improve the OS (P=0.17) or RFS (P=0.236) of Ph^-^B-ALL patients with *CDKN2A/B* deletion (**Figures 4G, H**). The survival of patients with single *CDKN2A/B* deletion was worse than that in patients without *CDKN2A/B* deletion, and the data are shown in **Supplementary Data 2**.

#### Ph-B-ALL Patients With the Simultaneous Deletions of IKZF1 & CDKN2A/B Had an Inferior Prognosis Compared With Patients Without Such Deletions

In Ph^-^B-ALL, 17 cases harbored the *IKZF1* and *CDKN2A/B* (*I&C*) deletions simultaneously, and these patients had worse OS and RFS than patients with no *I&C* deletion (N=64): 2-year OS: 34.3 ± 11.8% *vs*. 73.3 ± 5.5%, P=0.007; 2-year RFS: 33.3 ± 12.2% *vs*. 61.7 ± 6.3%, P=0.058 (**Supplementary Figures S3A, B**). The prognosis of patients with *I&C* deletion or single *CDKN2A/B* deletion was worse than that of patients without any gene deletions in Ph-B-ALL. The data are shown in **Supplementary Data 3**.

#### Analysis of the Prognosis Effect of Gene Deletion Combined With MRD Status

MRD results were obtained in 100 Ph^-^B-ALL patients. Among them, 38 patients were MRD-positive (22 patients received HSCT, 57.9%), and 62 patients were MRD-negative (22 patients underwent HSCT, 35.5%). Among 38 MRD positive patients, patients with gene deletions (N=27) had a worse prognostic tendency than those without any gene deletions (N=11). The 2-year OS was 25 ± 8.5% *vs*. 63.6 ± 14.5% (P=0.105), and 2-year RFS: 25.4 ± 8.5% *vs*. 45.5 ± 15% (P=0.277) (**Supplementary Figures S5A, B**). In MRD-negative Ph^-^B-ALL patients, the presence or absence of gene deletion had no significant effect on the prognosis (**Supplementary Figure S5C, D**).

In the 38 MRD-positive Ph^-^B-ALL patients, 20 cases had *CDKN2A/B* deletion (52.6%), 9 had *IKZF1* deletion, and 7 had *I&C* deletion. According to different gene deletions, the univariate survival of 38 patients was analyzed. The results showed that patients with *CDKN2A/B* deletion (N=20) had worse 2-year OS and RFS compared to patients without *CDKN2A/B* deletion (including patients with *IKZF1*deletion, N=18); the 2-year OS was 13.3 ± 8.1% *vs*. 60 ± 11.8% (P=0.007), and the 2-year RFS was 15 ± 8% vs. 50 ± 11.8% (P=0.084) (**Supplementary Figures S5E, F**). Among MRD-positive Ph^-^B-ALL patients, 21 cases received HSCT, including 12 cases with *CDKN2A/B* deletion. The 12 cases also observed worse survival than those without *CDKN2A/B* deletion (N=9); the 2-year OS was 22.2 ± 12.8% *vs*. 88.9 ± 10.5% (P=0.006), and the 2-year RFS was 27.3 ± 13.4% *vs*. 87.5.9 ± 11.7% (P=0.015) (**Supplementary Figures S5G, H**).

21/62 MRD-negative Ph^-^B-ALL patients had *CDKN2A/B* deletion (33.9%), 10 patients had *IKZF1* deletion, and 6 patients had *I&C* deletion. No survival differences were observed between gene deletion and no-gene deletion groups or from across different gene deletion groups (data not shown).

No obvious differences were noted in the survival with respect to *PAX5*, *ETV6*, *BTG1*, *EBF1*, or *RB1* deletions in both Ph^-^B-ALL patients.

### Prognosis of Gene Deletions in Ph^+^B-ALL

In the Ph^+^B-ALL cohort, the 2-year OS was 57.1 ± 5.4%, and the 2-year RFS was 47.8 ± 5.5%. Next, 70 patients had gene deletions, 15 patients had no gene deletions, and no statistical difference was observed in the survival between the two groups. In Ph^+^B-ALL patients, no clear prognostic significance of *IKZF1*, *CDKN2A/B, I&C*, *PAX5*, *ETV6*, *BTG1*, *EBF1*, or *RB1* deletion was recorded. MRD results were obtained in 72 Ph^+^B-ALL patients (MRD-negative: 48, MRD-positive: 24), and no significant prognostic significance was found in MRD subgroup analysis (**Table 3**).

**Table 3:** Univariate prognosis analysis of different gene deletions in Ph^+^B-ALL.

Ph^+^B-ALL (N=85)	2-year OS (%)	P Value	2-year RFS (%)	P Value
Gene deletion				
Yes (N=70)	60.9 ± 5.9	0.962	48.1 ± 6	0.946
No (N=15)	50.9 ± 13.3		46.7 ± 12.9	
CDKN2A/B del				
Yes (N=24)	50.9 ± 16.3	0.387	38.9 ± 10.5	0.644
No (N=61)	59.3 ± 9.5		50.8 ± 6.4	
IKZF1 del				
Yes (N=56)	64.3 ± 6.4	0.386	49.8 ± 6.7	0.287
No (N=29)	53.4 ± 9.6		43.9 ± 9.4	
I&C del				
Yes (N=19)	65.8 ± 9.9	0.387	38.9 ± 10.5	0.644
No (N=66)	56.7 ± 6.4		50.8 ± 6.4	
MRD				
positive (N=24)	54.6 ± 8.2	0.251	47.1 ± 8.1	0.419
negative (N=48)	73.5 ± 7.6		58.1 ± 8.6	

B-ALL, B-linage acute lymphoblastic leukemia; I&C, IKZF1 and CDKN2A/B; MRD, minimal residual disease; OS, overall survival; RFS, relapse free survival.

### COX Regression Analysis

The results of multivariate COX regression analysis were consistent with the univariate analysis. In Ph^-^B-ALL, the gene deletions constituted an independent prognostic factor after adjusted for age, WBC count and HSCT. *IKZF1* deletion in Ph^-^B-ALL patients had an independent prognostic significance in the non-HSCT group. *CDKN2A/B* showed independent prognostic significance in the overall Ph^-^B-ALL cohort: HSCT and non-HSCT groups. *I&C* deletion also had independent prognostic significance in the overall Ph^-^B-ALL cohort and non-HSCT group (**Table 4**). In addition, we also calculated MRD as the covariate in the COX regression, and found only CDKN2A/B deletion was the independent marker of OS and RFS after adjusting age, WBC count and MRD status (**Supplementary Table 2**). No independent prognostic factors were detected in Ph^+^B-ALL cohort.

**Table 4:** COX regression analysis of Ph^-^B-ALL patients with different gene deletions in different groups.

		Total^1^		HSCT^2^		NHSCT^2^		MRD+^3^	
		P	HR	P	HR	P	HR	P	HR
Gene del VS No del		0.004	2.313	0.149	2.134	0.038	2.033	0.131	2.342
	OS								
	RFS	0.017	1.862	0.519	1.257	0.022	2.261	0.181	1.917
IKZF1 del VS No IKZF1	OS	0.424	1.282	0.293	0.512	0.033	2.233	0.944	0.964
	RFS	0.653	1.158	0.517	0.736	0.261	1.644	0.738	0.847
CDKN2A/B del VS No C*		0.00	2.572	0.004	4.62	0.052	1.903	0.011	4.574
	OS								
	RFS	0.014	1.891	0.076	1.987	0.281	1.493	0.025	3.555
I&C Del VS No I &C	OS	0.01	2.657	0.22	2.621	0.018	2.82	0.332	1.889
	RFS	0.106	1.852	0.517	1.502	0.446	1.553	0.547	1.497
CDKN2A/B Only VS No I&C OS	OS	0.008	2.322	0.018	4.28	0.326	1.53	0.021	3.799
	RFS	0.069	1.747	0.184	1.807	0.476	1.391	0.023	3.715

^1^In total group, the values were adjusted by age, WBC and HSCT/NHSCT (HSCT was included in the model as a time-varying covariate); ^2^In HSCT/NHSCT group, the values were adjusted by age and WBC; ^3^In MRD+ group, the values were adjusted by age, WBC; *: No CDKN2A/B deletion.

B-ALL, B-linage acute lymphoblastic leukemia; HR, hazard ratio; HSCT, hematologic stem cell transplantation; I&C, IKZF1 and CDKN2A/B; MRD, minimal residual disease; NHSCT, non hematologic stem cell transplantation.

## Discussion

Despite overlapping morphological and immunophenotypic characteristics, adult and pediatric B-ALL have distinct molecular drivers and divergent treatment outcomes. Gene deletions have been closely associated with the prognosis of B-ALL with a primary focus on pediatric B-ALL (5, 21–23). However, the prognostic significance of gene deletions in Chinese adult B-ALL has not yet been well-established.

Herein, we used the MLPA method to detect gene deletions of 13 genes in 211 adult B-ALL patients. The results showed a high frequency of *IKZF1*, *CDKN2A/B*, and *PAX5* deletions in adult B-ALL patients. The incidence of *IKZF1* gene deletion in Ph^+^B-ALL patients (65.9%) was significantly higher than that in the Ph^-^B-ALL group (20.3%). In the current study, the incidence of *IKZF1+*other deletions is 21.3%, which is significantly higher than *IKZF1^PLUS^* that in pediatric BCP-ALL (6%) (13).

The frequency of *CDKN2A/B* deletion was significantly higher in the Ph^-^B-ALL group (39.7%) than in the Ph^+^B-ALL group (24.7%). *CDKN2A/B* deletion occurred in about 36-45% pediatric B-ALL patients (10, 24, 25), which is similar to that in adult B-ALL.

In the prognostic analysis of Ph^-^B-ALL patients, the prognosis of patients with gene deletions was significantly worse than in patients without any gene deletion. *IKZF1* is one of the most common deleted gene in B-ALL (14), and its prognostic significance is controversial. Dorge et al. (15–17) considered that *IKZF1* gene deletion has a significant impact on the prognosis of pediatric B-ALL. It can be incorporated in the risk stratification system and defined as the high-risk group. However, Stanulla et al. (13) and other studies (18, 19) suggested that although the prognosis of pediatric B-ALL with *IKZF1* deletion was poor, the OS of pediatric B-ALL with *IKZF1* deletion was satisfactory, and hence, its prognostic significance was limited. Zhang et al. (26) conducted a meta-analysis on eight national and international studies about the prognosis of *IKZF1* gene deletion in adult B-ALL and speculated that *IKZF1* gene deletion indicates poor prognosis; however, this prognostic significance only exists in patients with Ph^-^B-ALL. Moreover, as Messina et al. reported, the outcome of *IKZF1* deletion did not differ from that of *IKZF1*-*WT* patients both in pediatric and adult B-NEG ALL (B-ALL patients negative for the recurrent fusion transcripts, *BCR-ABL*, *ETV6-RUNX1, TCF3-PBX1, KMT2A*-rearrangement) (27). In the current analysis, which including 126 Ph^-^B-ALL patients, *IKZF1* deletion was found to be related to poor OS in the group of patients who did not receive HSCT (2-year OS: 17.9 ± 11% *vs*. 62.6 ± 6.3%, P=0.037). Consequently, patients with *IKZF1* deletion would be recommended to undergo HSCT, which demonstrated superior survival benefit compared to chemotherapy alone.

The prognostic significance of *IKZF1* deletion in Ph^+^B-ALL was also not consistent: among the 83 adult Ph^+^B-ALL cases reported by Martinelli et al. (5), DFS was shorter in patients with *IKZF1* deletion compared to those without *IKZF1* deletion. However, in another study by Yao et al. on 43 Ph^+^B-ALL patients (22), those with *IKZF1* deletion did not show any significant differences in non-relapse mortality, leukemia-free survival, or survival with patients without the deletion. In report of Pfeifer et al. (10), the prognostic significance of *IKZF1* was weaker than *CDKN2A/B*, and *IKZF1* deletion alone actually had a significant lower risk for relapse than with other deletions. In the current study, *IKZF1* deletion did not show any prognostic significance in Ph^+^B-ALL patients.

The prognostic significance of *CDKN2A/B* deletion is controversial in pediatric B-ALL (24, 25, 28). In the study by Mirebeau et al. (29), *CDKN2A/B* deletions coincided with other HR factors in pediatric B-ALL but did not affect prognosis. However, Heyman et al. (30) found that *CDKN2A/B* was significantly associated with poor prognosis in 79 pediatric B-ALL patients. In study of Messina et al., *CDKN2A/B* deletion had impact of DFS in only adult cohort of B-NEG ALL (26). Owing to its high deletion incidence in adult B-ALL, we also analyzed the prognosis of patients with *CDKN2A/B* gene deletion and found that the deletion was an independent poor prognostic indicator in the Ph^-^B-ALL cohort, regardless of HSCT, and the single *CDKN2A/B* deletion was also associated with poor prognosis in each subgroup of Ph^-^B-ALL. In another study by Pfeifer et al. (10), *CDKN2A/B* deletion was detected in 45% Ph^+^B-ALL cases and indicative poor OS and DFS with a short remission duration. Nonetheless, in this study, the proportion of *CDKN2A/B* deletion in Ph^+^B-ALL was low (28.2%), and no statistical prognostic significance was detected in any subgroup.

Stanulla et al. (13) defined *IKZF1^plus^* and proposed it as an MDR-dependent, poor prognostic profile in pediatric B-ALL. In the current study, compared to *PAX5* and *PAR1, IKZF1* deletion occurred more frequently with *CDKN2A/B* deletion in adult B-ALL patients, especially in Ph^-^B-ALL patients. In this cohort, despite the small population size, patients with simultaneous deletion of *I&C* had worse OS and RFS than those without the deletion. Hence, we speculated that the prognostic significance of *I&C* in adult Ph^-^B-ALL is similar to that of *IKZF1^plus^* in pediatric B-ALL, and hence, in this study, the prognostic significance of *IKZF1*+other gene deletions was not analyzed separately. In future studies, we would need to expand the sample size to substantiate these findings. In Ph^+^B-ALL, the prognostic significance of *I&C* deletions was not detected.

MRD is a critical index to evaluate the prognosis of ALL patients (31–36). We analyzed the prognostic significance of gene deletions stratified by MRD status. In Ph^-^B-ALL patients with negative MRD, the gene deletion had no obvious effect on survival. In 38 MRD-positive Ph^-^B-ALL patients, those with gene deletions had worse OS and RFS than those without any gene deletions, indicating that gene deletions affected the prognosis of MRD-positive Ph^-^B-ALL patients. We also found that the incidence of *CDKN2A/B* deletion was significantly higher (52.6% *vs*. 33.9%, P=0.064) in these patients, and it was a poor prognostic factor in MRD-positive Ph^-^B-ALL, regardless of HSCT. However, our data demonstrated that MRD-positive patients without *CDKN2A/B* deletion could benefit from allo-HSCT, as the positive MRD is an adverse risk factor. On the other hand, the MRD-negative Ph^-^B-ALL cohort did not show any survival differences in different gene deletion groups, indicating that patients carrying *CDKN2A/B* deletion with a negative MRD could be potentially spared from HSCT. Taken together, these data suggested an MRD-dependent prognostic role of *CDKN2A/B* in allo-HSCT decision for Ph^-^B-ALL patients. In Ph^+^B-ALL patients, no significant effect of gene deletions was found on survival irrespective of the MRD status. Thus, in Chinese adult Ph^+^B-ALL, the Ph chromosome, not gene deletions plays a significant role in determining the prognosis of patients.

In conclusion, we proved that gene deletions play a major role in the prognosis of Chinese adult B-ALL patients, and we did the analyses in allotransplanted and MRD subsets in the current study. The overall survival of Ph^-^B-ALL patients with gene deletion is worse than that of patients without any gene deletion, Ph^+^B-ALL patients is not affected by gene deletion. In Ph^-^B-ALL, patients with *IKZF1* or *CDKN2A/B* deletion showed poor prognosis; allo-HSCT could improve the OS in patients with *IKZF1* deletion but could not improve the OS in patients with *CDKN2A/B* deletion. *CDKN2A/B* deletion also has a negative impact on survival only in MRD positive Ph^-^B-ALL patients

## Data Availability Statement

The datasets presented in this study can be found in online repositories. The names of the repository/repositories and accession number(s) can be found in the article/**Supplementary Material**.

## Ethics Statement

The studies involving human participants were reviewed and approved by the ethics committee of Institute of Hematology and Blood Diseases Hospital, Chinese Academy of Medical Sciences. Written informed consent to participate in this study was provided by the participants’ legal guardian/next of kin.

## Author Contributions

YM and JXW contributed to the study design. YM and QF were involved in analyzing and interpreting the data. QF wrote the article. YF and QF were involved in the statistical analysis. All authors were involved in the collection and assembly of clinical data. YS, XG, JW, KL, QH, YL, HW, GZ, YTL, BG, YW, CZ, DL, BL, SW, and RG provided the study materials or patients. All authors contributed to the article and approved the submitted version.

## Fuinding

This study was supported by National Key Research and Development Program of China (2019YFC0840605), CAMS Innovation Fund for Medical Sciences (2018-I2M-AI-017), and National Science and Technology Major Project (2017ZX09304024).

## Conflict of Interest

The authors declare that the research was conducted in the absence of any commercial or financial relationships that could be construed as a potential conflict of interest.
