# Supplementary material for: Gene Deletions and Prognostic Values in B-Linage Acute Lymphoblastic Leukemia
Source: Front Oncol. 2021 Jun 2;11:677034. doi: 10.3389/fonc.2021.677034 (PMC8206559; doi:10.3389/fonc.2021.677034)
Supplement: Supplementary file 1 [file DataSheet_1.pdf]

Supplementary Data 1.

Among 211 patients, 9 patients didn't achieve CR, they were all died in 1 year. 8/9 patients of them harbored gene deletions (Ph$^-$B-ALL: 6, Ph$^+$B-ALL: 2). Among patients with gene deletion, 5 were *IKZF1* deletion (Ph$^-$B-ALL: 3, Ph$^+$B-ALL: 2), 4 were *CDKN2A/B* deletion (Ph$^-$B-ALL: 3, Ph$^+$B-ALL: 1); 2 were Ph$^-$B-ALL with *IKZF1* and *CDKN2A/B* deletion, 3 were Ph$^-$B-ALL with *PAX5* deletion.

There were 6 patients who received allo-HSCT at CR2, and they were all Ph$^-$B-ALL patients, 2 carried gene deletion (*IKZF1* deletion: 1, *CDKN2A/B* deletion: 1); 3 of them were SR group.

Supplementary Data 2.

The prognosis of patients with single CDKN2A/B deletion was worse than those without CDKN2A/B deletion.

There were 36 Ph$^-$B-ALL patients had single *CDKN2A/B* deletion (without *IKZF1* deletion), these patients got worse OS and RFS than those without *CDKN2A/B* and *IKZF1* deletion (N= 64), the 2-year OS were 42.9±8.5%VS 73.3±5.5%，P=0.011; the 2-year RFS were 42.7±8.4% vs. 61.7±6.3%，P=0.049 (Figure S2A and B).

Allo-HSCT did not significantly improve the prognosis of Ph$^-$B-ALL patients with such deletion. In HSCT group, patients with single *CDKN2A/B* deletion (N=20) vs. no such deletion patients (N=18), the 2-year OS were 35.4%±11% vs. 79.3%±9. 2%，P=0.021; the 2-year RFS

were 47.4±11.5% vs. 85.7±9.4%, P=0.035 (Figure S2E and F). In non-HSCT group, patients with single *CDKN2A/B* deletion (N=16) vs. no such deletion patients (N=46), the 2-year OS were 18.7±14.8% vs. 62.4%±7.5%, P=0.05; the 2-year RFS were 36.5±12.3% vs. 54.6±7.4%, P=0.123 (Figure S2C and D). We also compared the survivals between Ph^-B-ALL patients with single *CDKN2A/B* deletion who received HSCT and who did not receive HSCT, and no significant difference was found (2-year OS: 47.1%±11% vs. 46.7±12.9%, P=0.346; 2-year RFS: 47.4±11.5% vs. 36.5±12.3%, P=0.308, Figure S2G and H).

In Ph^+B-ALL patients, the prognosis was not affected by the single *CDKN2A/B* gene deletion, regardless of HSCT.

Supplementary Data 3.

The prognosis of patients with CDKN2A/B & IKZF1 deletion or single CDKN2A/B deletion was worse than patients without any gene deletions in Ph-B-ALL.

In Ph^-B-ALL, we compared the survival in the below three groups: none gene deletion group (N=52), *I&C* deletion group (N=17) and single *CDKN2A/2B* deletion group (N=36). Patients with only *IKZF1* deletion were not included because of the small case number. The none gene deletion group had a better OS and RFS than the other two groups, and prognosis between *I&C* deletion group and single *CDKN2A/2B* deletion group were similar. None gene deletion group vs. *I&C* group vs. single

CDKN2A/B group: 2-year OS: 72.1±6.4% VS 34.3±11.8 vs. 46.5±8.4, 1 vs. 2, P=0.004, 1 vs. 3, P=0.007; 2 vs. 3, P=0.573; 2-year RFS: 62±6.9% vs. 33.3±12.2 vs. 42.7±8.4%, 1 vs. 2, P=0.06, 1 vs. 3, P=0.055, 2 vs. 3, P=0.665 (Figure S4A and B). Other types of gene deletions were not compared because of the small case number.

Table S1. MRD status in different gene deletion types.

		MRD+(62)	MRD-(110)	P value
IKZF1 Del(66)	Total	30	36	0.043
	Ph+	21	26	0.843
	Ph-	9	10	
IKZF1+other del(36)	Total	15	21	0.43
	Ph+	7	15	0.133
	Ph-	8	6	
CDKN2A/B(62)	Total	26	36	0.37
	Ph+	6	15	0.127
	Ph-	20	21	
I&C(29)	Total	11	18	0.817
	Ph+	4	12	0.111
	Ph-	7	6	
PAX5(44)	Total	16	28	0.959
	Ph+	7	12	0.954
	Ph-	9	16	

Table S2. COX regression analysis of Ph^{-}B-ALL patients including covariate: CDKN2A/B, MRD, WBC count, age and HSCT.

		P	HR
WBC count	OS	0.01	1.005
	RFS	0.012	1.003
Age	OS	0.763	1.003
	RFS	0.185	1.014
MRD	OS	0.00	6.213
	RFS	0.00	4.712
CDKN2A/B del	OS	0.05	2.561
	RFS	0.059	1.765
HSCT	OS	0.003	0.33
	RFS	0.065	0.543

Figure Legend:

Figure S1. Survival of 211 B-ALL patients, and in different Ph group by Kaplan-Meier plots

A-B The 2-year OS and RFS of 211 B-ALL patients.

C-D The 2-year OS and RFS of 211 patients in Ph-B-ALL group and Ph+B-ALL group.

Figure S2: Effect of *CDKN2A/B* deletion on survivals of Ph-B-ALL patients by Kaplan-Meier plots

A-B The 2-year OS and RFS of Ph-B-ALL patients who carried single *CDKN2A/B* deletion vs. no *CDKN2A/B* deletion.

C-D The 2-year OS and RFS of Ph-B-ALL patients who carried single *CDKN2A/B* deletion vs. no *CDKN2A/B* deletion in no HSCT group.

E-F The 2-year OS and RFS of Ph-B-ALL patients who carried single *CDKN2A/B* deletion vs. no *CDKN2A/B* deletion in HSCT group.

G-H The 2-year OS and RFS of Ph-B-ALL patients with single *CDKN2A/B* deletion who received HSCT VS no HSCT.

Figure S3: Effect of *I&C* deletion on survivals of Ph-B-ALL patients by Kaplan-Meier plots

A-B The 2-year OS and RFS of Ph-B-ALL patients who carried *I&C* deletion vs. no *I&C* deletion.

Figure S4: Survivals of Ph-B-ALL patients with *I&C* deletion or single *CDKN2A/B* deletion vs. patients without any deletions by Kaplan-Meier plots

A-B The 2-year OS and RFS of Ph-B-ALL patients who carried *I&C* deletion vs. single CDKN2A/B deletion vs. patients without any deletions.

Figure S5: Effect of MRD and gene deletion on survivals of Ph-B-ALL patients by Kaplan-Meier plots

A-B The 2-year OS and RFS of MRD positive (MRD+) Ph-B-ALL patients who carried gene deletions vs. no gene deletions.

C-D The 2-year OS and RFS of MRD negative (MRD-) Ph-B-ALL patients who carried gene deletions vs. no gene deletions.

E-F The 2-year OS and RFS of MRD positive (MRD+) Ph-B-ALL patients who carried *CDKN2A/B* deletion vs. no *CDKN2A/B* deletion.

G-H The 2-year OS and RFS of MRD positive (MRD+) Ph-B-ALL patients who carried *CDKN2A/B* deletion vs. no *CDKN2A/B* deletion in HSCT group.

Figure S1.

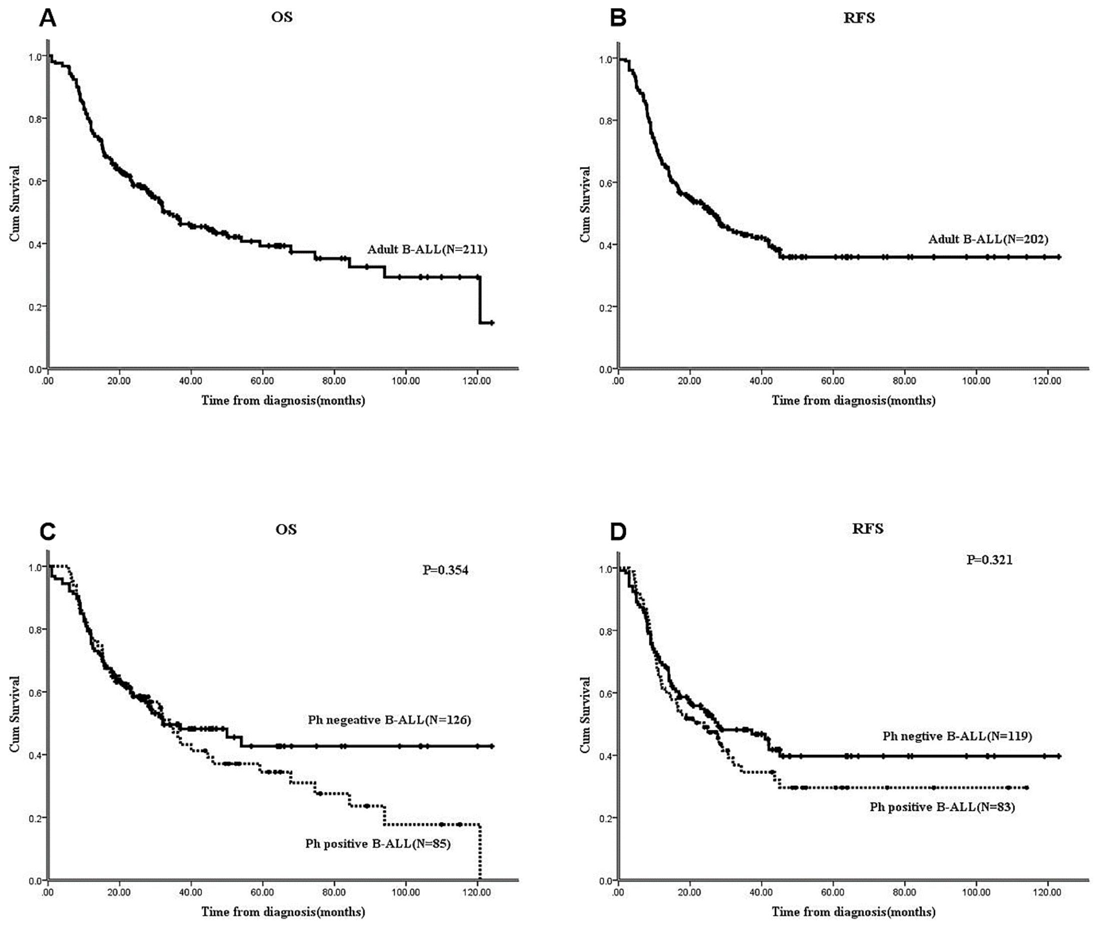
A
OS
Adult B-ALL(N=211)
B
RFS
Adult B-ALL(N=202)
C
OS
P=0.354
Ph negeative B-ALL(N=126)
Ph positive B-ALL(N=85)
D
RFS
P=0.321
Ph negtive B-ALL(N=119)
Ph positive B-ALL(N=83)
Cum Survival
Time from diagnosis(months)

Figure S2.

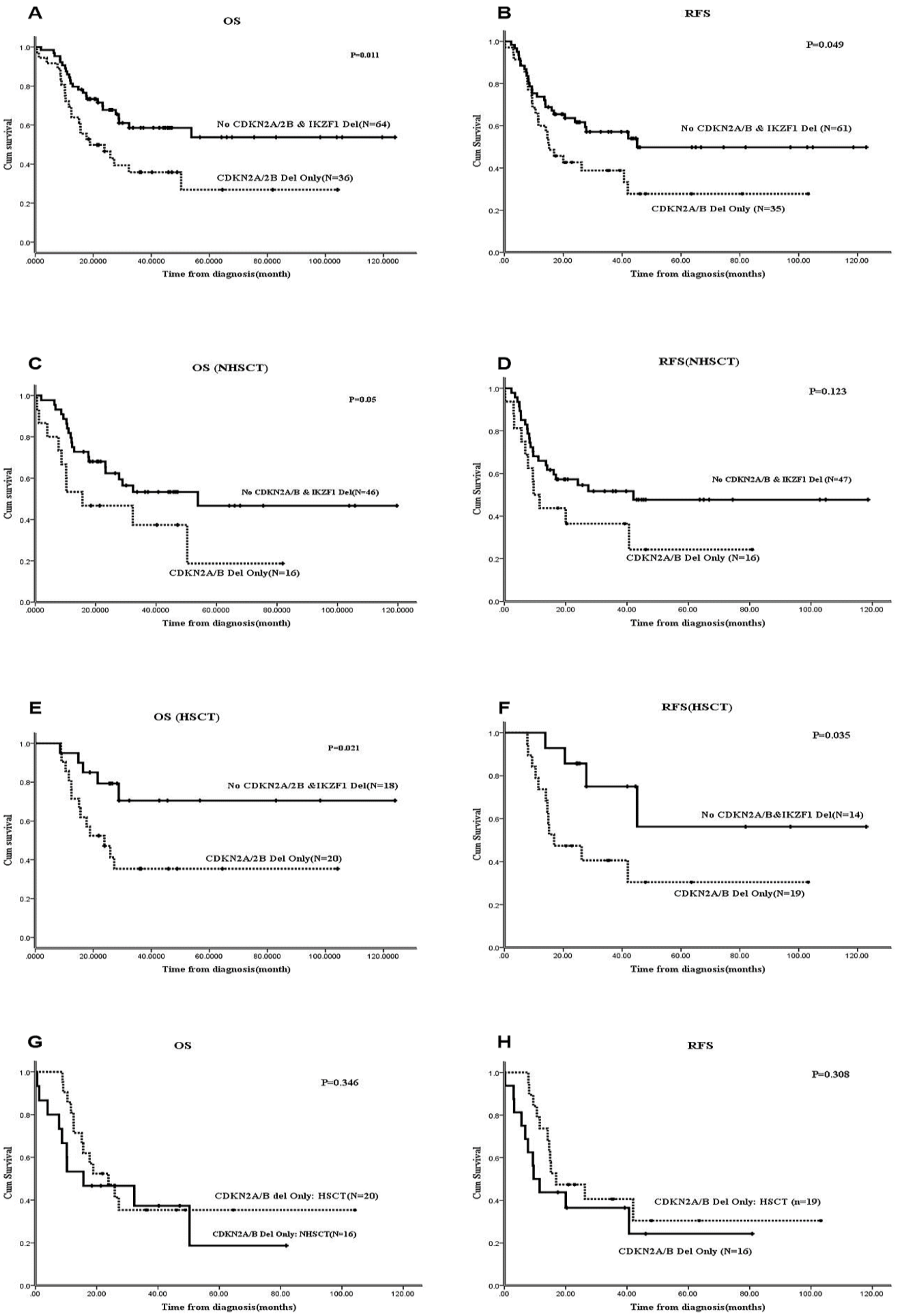
A
OS
P=0.011
No CDKN2A/2B & IKZF1 Del(N=64)
CDKN2A/2B Del Only(N=36)
Cum survival
Time from diagnosis(month)
B
RFS
P=0.049
No CDKN2A/B & IKZF1 Del (N=61)
CDKN2A/B Del Only (N=35)
Cum Survival
Time from diagnosis(months)
C
OS (NHSCT)
P=0.05
No CDKN2A/B & IKZF1 Del(N=46)
CDKN2A/B Del Only(N=16)
Cum survival
Time from diagnosis(month)
D
RFS(NHSCT)
P=0.123
No CDKN2A/B & IKZF1 Del (N=47)
CDKN2A/B Del Only (N=16)
Cum Survival
Time from diagnosis(months)
E
OS (HSCT)
P=0.021
No CDKN2A/2B &IKZF1 Del(N=18)
CDKN2A/2B Del Only(N=20)
Cum survival
Time from diagnosis(month)
F
RFS(HSCT)
P=0.035
No CDKN2A/B&IKZF1 Del(N=14)
CDKN2A/B Del Only(N=19)
Cum Survival
Time from diagnosis(months)
G
OS
P=0.346
CDKN2A/B del Only: HSCT(N=20)
CDKN2A/B Del Only: NHSCT(N=16)
Cum Survival
Time from diagnosis(months)
H
RFS
P=0.308
CDKN2A/B Del Only: HSCT (n=19)
CDKN2A/B Del Only (N=16)
Cum Survival
Time from diagnosis(months)

Figure S3.

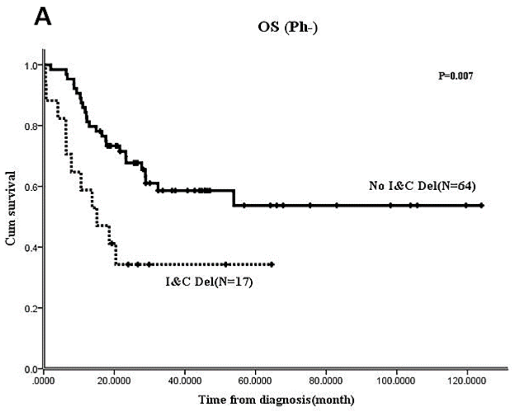

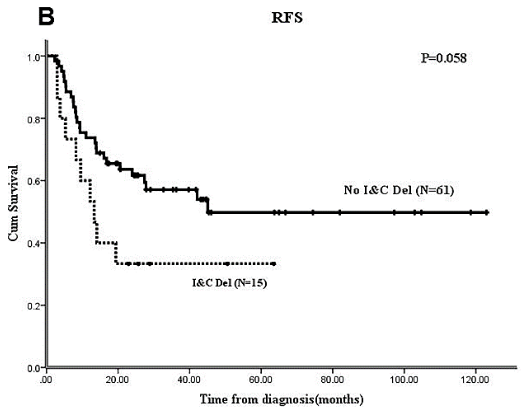

Figure S4.

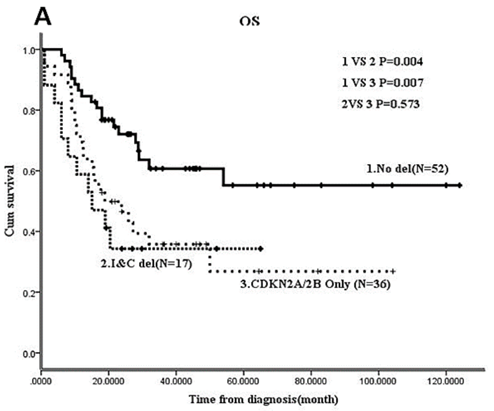

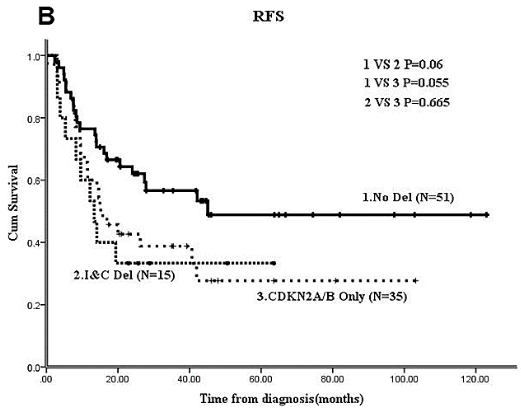

Figure S5

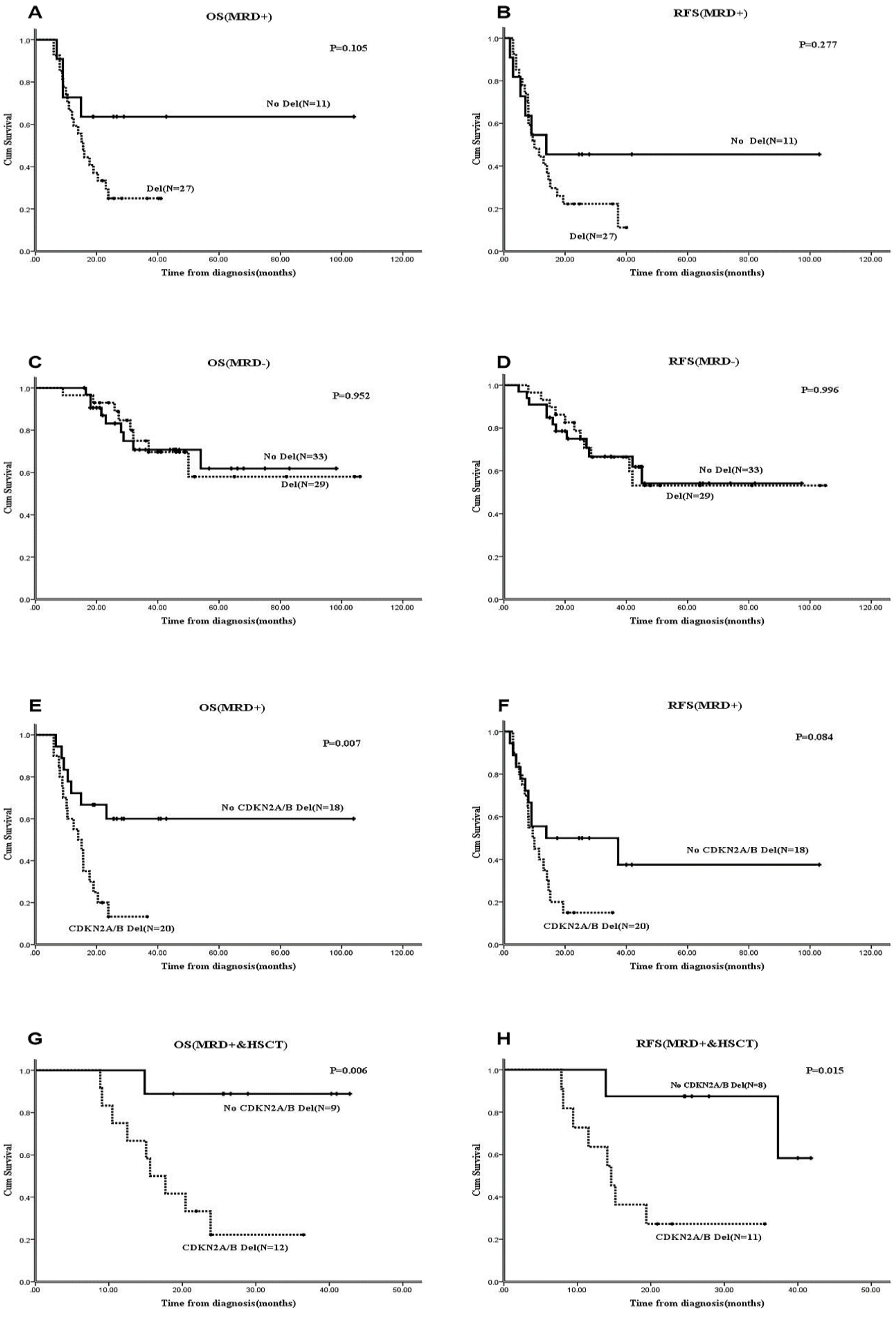
A
OS(MRD+)
P=0.105
No Del(N=11)
Del(N=27)
B
RFS(MRD+)
P=0.277
No Del(N=11)
Del(N=27)
C
OS(MRD-)
P=0.952
No Del(N=33)
Del(N=29)
D
RFS(MRD-)
P=0.996
No Del(N=33)
Del(N=29)
E
OS(MRD+)
P=0.007
No CDKN2A/B Del(N=18)
CDKN2A/B Del(N=20)
F
RFS(MRD+)
P=0.084
No CDKN2A/B Del(N=18)
CDKN2A/B Del(N=20)
G
OS(MRD+&HSCT)
P=0.006
No CDKN2A/B Del(N=9)
CDKN2A/B Del(N=12)
H
RFS(MRD+&HSCT)
P=0.015
No CDKN2A/B Del(N=8)
CDKN2A/B Del(N=11)
Cum Survival
Time from diagnosis(months)
